# Early Detection of Recurrence and Progress Using Serum Steroid Profiling by LC–MS/MS in Patients with Adrenocortical Carcinoma

**DOI:** 10.3390/metabo14010020

**Published:** 2023-12-28

**Authors:** Otilia Kimpel, Barbara Altieri, Ulrich Dischinger, Carmina Teresa Fuss, Max Kurlbaum, Martin Fassnacht

**Affiliations:** 1 Division of Endocrinology and Diabetes, Department of Medicine, University Hospital, University of Würzburg, 97080 Würzburg, Germany; altieri_b@ukw.de (B.A.); fuss_c@ukw.de (C.T.F.); kurlbaum_m@ukw.de (M.K.); fassnacht_m@ukw.de (M.F.); 2 Core Unit Clinical Mass Spectrometry, Central Laboratory, University Hospital Würzburg, 97080 Würzburg, Germany; 3 Comprehensive Cancer Center Mainfranken, University of Würzburg, 97080 Würzburg, Germany

**Keywords:** adrenal cancer, follow-up, steroid measurement, liquid chromatography–tandem mass spectrometry (LC–MS/MS)

## Abstract

Serum liquid chromatography–tandem mass spectrometry (LC–MS/MS) steroid profiling is used for the diagnosis of adrenocortical carcinoma (ACC). Guidelines recommend endocrine work-up in addition to radiological imaging for follow-up in ACC, but data on this topic are scarce. Patients were included in this retrospective study if pre-therapeutic hormone values, regular tumour evaluation by imaging, steroid measurements by LC–MS/MS, and details on therapies were available. The utility of steroid profiles in detecting recurrence or disease progression was assessed, whereby “endocrine progress” was defined by an elevation of at least 3 of 13 analysed hormones. Cohort A included 47 patients after R0 resection, of whom 15 experienced recurrence and 32 did not. In cohort B, 52 patients with advanced disease (including 7 patients of cohort A with recurrence) could be evaluated on 74 visits when progressive disease was documented. In 20 of 89 cases with documented disease progression, “endocrine progress” was detectable prior to radiological progress. In these cases, recurrence/progression was detected at a median of 32 days earlier by steroid measurement than by imaging, with 11-deoxycortisol and testosterone being the most sensitive markers. Notably, these patients had significantly larger tumour burden. In conclusion, steroid profiling by LC–MS/MS is of value in detecting recurrent/progressive disease in ACC.

## 1. Introduction

Adrenocortical carcinoma (ACC) is a rare but aggressive endocrine tumour, which leads to relevant hormone excess in 50–60% of patients [1,2,3,4]. The majority of patients presenting with a hormonal excess suffer from cortisol or androgen hypersecretion alone or in combination [5,6]. Moreover, elevated steroid hormone precursors, which are typical for a disorganized steroidogenesis and, thus, almost pathognomonic for ACC, are detectable in the vast majority of patients with ACC [1,7,8,9,10,11]. Some previous studies demonstrated that steroid profiling may distinguish ACC from adrenocortical adenoma. This examination can be performed best by liquid chromatography–tandem mass spectrometry (LC–MS/MS) either in serum [12,13,14] or urine [10,15,16,17,18,19]. Suzuki et al. demonstrated a distinction between cortisol-producing adrenocortical carcinoma and cortisol-producing adenoma by using serum LC–MS/MS and urine GC–MS measurements. They observed significantly higher levels of steroid precursors in ACC compared to cortisol-producing adenoma [20]. Both studies by Taylor et al. and Schweitzer et al. indicated significantly increased steroid precursors and androgens as distinctive for the primary diagnosis of ACC [12,14]. In urine, the value of steroidobolomics was already demonstrated in 2011 [10] and recently confirmed in a large validation study [18].

Currently, the management of patients with ACC includes surgery followed by adjuvant therapies (e.g., mitotane, radiotherapy or platin-based therapy) or active surveillance in a subset of low-risk patients with localized disease [1,7,21,22,23,24,25,26,27,28,29,30]. Treatment of recurrent disease is usually individualized [31] whereas in advanced disease, mitotane ± chemotherapy are the cornerstones of therapy [32,33,34]. However, local therapies can be interesting treatment options [35,36,37,38].

Both ACC guidelines recommend a hormonal evaluation for suspected ACC including serum cortisol, aldosterone, 17-hydroxyprogesterone, dehydroepiandrosterone sulfate (DHEAS), androstenedione, testosterone and 17-beta-estradiol and, if available, 11-deoxycortisol/adrenocortical steroid hormone precursors [1,7].

There was always the perception that these steroids could also serve as useful biomarkers for the follow-up of patients in addition to the recommended regular radiological imaging. Since laboratory work-up is less stressful for patients and less expensive than imaging, it could be performed more frequently. Such a procedure may facilitate an earlier detection of recurrences or progression to allow an earlier treatment modification with the goal to improve survival. Thus, the guidelines recommend regular screening for hormone secretion during follow-up [1,7]. However, until now, only one single study assessed the utility of steroid hormone profiling in recurrence detection with GC–MS [15]. This study utilizing 24 h urine analyses by GC–MS could demonstrate detection of recurrence by steroid profiling two months earlier in 22% to 39% of patients in comparison to radiological imaging [15]. However, collection of 24 h urine samples is cumbersome for the patients and the GC–MS method used is time consuming and only very few centres have access to this method. In contrast, serum samples are easier to collect and LC–MS/MS is increasingly used in clinical practice.

In the present study, we aimed to investigate the utility of serum LC–MS/MS steroid profiling in detecting a recurrent or progressive disease earlier than with radiological imaging.

## 2. Methods

### 2.1. Patients

This study was part of the ENSAT registry study (www.ensat.org/registry) (accessed on 1 January 2016) in our centre in Würzburg. It was approved by our local ethics committee and all patients provided written informed consent. Patients with a primary diagnosis of ACC since 2016 were included if the following information was available: pre-therapeutic hormone evaluation, imaging for tumour evaluation every 2–4 months, details on therapies and follow-up, and regular steroid measurements by LC–MS/MS. Follow-up for this study was closed in May 2023. Patients with a lack of relevant information on hormone evaluation, without LC–MS/MS measurements or radiological imaging were excluded.

Demographic, clinical, and histological parameters (sex, age at diagnosis, tumour size, evidence of hormonal excess, ENSAT tumour stage [39], information on therapies during follow-up, results of LC–MS/MS measurements, and tumour evaluation during follow-up) were retrieved from the ENSAT ACC registry and medical records. All histological diagnoses were confirmed by experienced pathologists. Tumour staging at diagnosis was based on imaging studies and by the findings during surgery and pathological examination.

**Figure 1:**
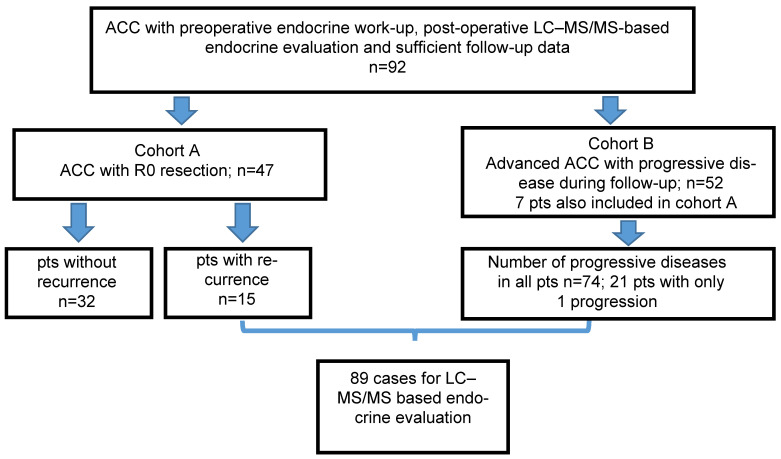
Patient selection and final cohort. pts = patients, *n* = number.

Patients were divided in two groups: cohort A consisted of patients with R0 resection and cohort B of patients with advanced ACC with progressive disease during follow-up (see Figure 1). In the latter group, every visit with documented progress was analysed separately if all required data were available.

### 2.2. Methods and Time Interval for Imaging during Follow-Up

Follow-up imaging was regularly performed in each patient according to current ACC guidelines [1,7]. Thoraco-abdominal imaging was performed either with a thoracic and abdomen computed tomography (CT) scan, thoracic CT and abdominal magnetic resonance imaging (MRI), or with 18-fluoro-2-deoxyglucose (F-18-FDG)-positron emission tomography scan including a full diagnostic computed tomography scan (F-18-FDG-PET/CT).

### 2.3. LC–MS/MS Measurements

All steroid measurements were part of the predefined diagnostic work-up in our centre. Accordingly, the measurements were performed within a few days (regularly within less than one week). Measurements were performed with a liquid chromatography–tandem mass spectrometry system (QTRAP 6500+, SCIEX^®^, Framingham, MA, USA) including an Agilent 1290 HPLC (G4226A autosampler, infinityBinPump, G1316C column-oven, G1330B thermostat, Santa Clara, CA, USA). The MassChrom-Steroids in Serum/Plasma^®^ IVDR conform kit (Chromsystems^®^, Gräfelfing, Germany) was used, allowing the quantification of 15 steroid hormones in the positive MRM-Mode (aldosterone, DHEAS in the negative mode), via corresponding isotope-labelled standards according to the manufacturer’s instructions. The method was verified following the regulation (EU) 2017/746 of the European parliament and of the council, Annex 1 (5 April 2017) [40]. Pre-analytic preparation is based on several steps including separation, cleaning and concentrating of the samples (all steps are described in detail in the manufacturer’s instructions, IM 72072, 09/2022 R3.1, p. 18) [41]. Accordingly, 500 μL of serum was processed by off line solid phase extraction and finally 15 μL was used for analysis. For quantitative analysis of raw data, the Analyst^®^ Software (1.6.3) via 6-point calibration and 1/x weighting was used. Commercial quality controls and periodic participation in ring trails ensured the correctness of measurements for the analytes (lower limit of quantification, LLOQ): aldosterone (10 ng/L), androstenedione (0.022 µg/L), 11-deoxycortisol (0.03 µg/L), 11-deoxycorticosteron (0.023 µg/L), 21-deoxycortisol (0.027 µg/dL), cortisol (0.152 µg/dL), cortisone (0.148 µg/L), corticosterone (0.175 µg/L), dehydroepiandrosterone (DHEA) (0.229 µg/L), dehydroepiandrosterone sulfate (DHEAS) (2.44 µg/dL), dihydrotestosterone (DHT) (42 ng/L), oestradiol (30 ng/L), progesterone (0.03 µg/L), 17-hydroxyprogesterone (17-OHP) (0.023 µg/L), and testosterone (5 ng/L).

The glucocorticoids cortisol and cortisone were excluded as these are uninterpretable in mitotane-treated ACC patients due to a high-dose glucocorticoid replacement and the strong induction of the cortisol metabolizing enzyme CYP3A4 by mitotane [42].

### 2.4. Outcome Assessment

Prior to any analysis, we defined as evidence for biochemical progression or recurrence (“endocrine progress”) when a minimum of three steroids measured by LC–MS/MS at a given date were above 1.5-fold of the upper limit of the age- and sex-adjusted normal range. These cut-offs were chosen based on our clinical experience to avoid too many false-positive results due to biological and clinical variability. In post-hoc analyses, we also evaluated if other cut-offs would lead to better results, but this was not the case and, therefore, we kept the predefined definition. Recurrence and progression were verified for each case based on routine radiological assessment. Each documented recurrence and progression were counted as an individual case. Patients with more than one recurrence or progression were analysed several times.

### 2.5. Statistical Analysis

Data analysis and graphic representation was completed using SPSS version 26 (IBM SPSS Statistics) and GraphPad Prism (version 10.0.2, La Jolla, CA, USA). Data are summarized as median (interquartile range) values unless otherwise stated.

Continuous variables were reported as median with lower and upper quartile (Q1–Q3) if not otherwise specified, whereas categorical variables were reported as numbers and percentages. Values of steroid hormones were normalized to the upper limit of the reference range in sex-matched healthy controls. A Two-sided *t* test or Mann–Whitney test and ANOVA or Kruskall–Wallis test were used to compare continuous variables, as appropriate, while the chi-square (χ²) test was used to compare categorical variables. All reported *p* values are two-sided. *p* values of less than 0.05 were considered to indicate statistical significance.

## 3. Results

### 3.1. Patients’ Characteristics

The total cohort consisted of 92 patients. Cohort A had 47 patients with R0 resection, of whom 15 patients developed recurrence during follow-up and 32 patients remained without recurrence. Cohort B contained 52 patients with advanced ACC and progressive disease (including 7 patients of cohort A with recurrence). For patients with progressive disease occurring more than once, each of these dates was investigated separately, resulting in a number of 74 cases with progressive disease, leading to total 89 cases with recurrence or progress documented by imaging (Figure 1). Patients’ characteristics are given in Table 1. All patients had a histological confirmed ACC and had a preoperative hormone evaluation. Most of them presented with a glucocorticoid and androgen excess at first diagnosis. All patients had routine imaging with CT, MRI, and/or FDG/PET–CT every two to four months. Steroid hormones were measured at every time point of imaging and in some patients in between by LC–MS/MS.

Age, sex, concomitant therapies, surgical approach, imaging method, median time to recurrence or progress, and tumour burden at the time of recurrence or progress did not differ significantly between the three groups, whereas resection status, ENSAT stage, preoperative hormone excess, Ki67 index, primary tumour size, and follow-up time were significantly different between the groups (see Table 1).

### 3.2. Detection of a Recurrence and Progressive Disease

**Figure 2:**
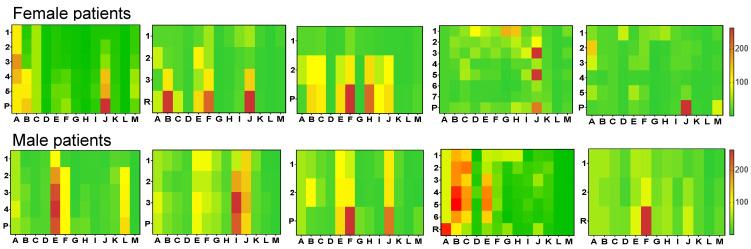
Heat-map visualization of longitudinal serum steroid profile results in 5 female and 5 male patients who developed recurrent or progressive disease during follow-up. 5 female patients, from left to right, including 3 cases in whom steroid profiling already indicated progressive disease before this was detected at imaging, one patient with a false-positive elevation, and one patient without any hormone elevation ahead of imaging. 5 male patients from left to right including 3 cases with representative hormone elevation before recurrence or progressive disease, one patient with a false-positive elevation, and one patient without any hormone elevation. Y axis shows longitudinal hormone measurements. Last measurement indicated the detected R recurrence or P progressive disease by radiologic imaging. X axis shows steroids. A: oestrogen; B: testosterone; C: dihydrotestosterone; D: progesterone, E: 17-OHP; F: androstenedione; G: DHEAS; H: DHEA; I: aldosterone, J: 11-deoxycortisol; K: 11-deoxycorticosterone; L: 21-deoxycortisol; M: corticosterone. The colour code on the right side indicates the degree of hormone excess in %, 100 indicates the upper limit of normal of each hormone (in a sex- and age-adjusted manner).

Median time to recurrence (defined by radiologic evaluation) was 360 days in patients after R0 resection (*n* = 15), whereas time to progression was 303 days in 74 evaluated cases. In two (13.3%) of 15 patients in cohort A and in 18 (24.3%) of 74 cases in cohort B at least three steroid hormones were already elevated before the recurrence or progressive disease could be detected by radiologic imaging. Representative examples are given in Figure 2. In these 20 cases, recurrence or disease progression was detected after a median of 301 (118–626) days with radiologic imaging whereas the endocrine alterations were already visible after 234 (89–540) days (*p* < 0.001). Recurrence and progression were detected in a median of 32 (27–65) days earlier by steroid measurement in comparison to radiologic imaging. If we considered only two elevated steroid hormones as sufficient to define “endocrine progress”, we could identify four more cases, in which “endocrine progress” was detectable before radiologic imaging confirmed this progress. The precursor steroid 11-deoxycortisol and testosterone were elevated in the majority of these cases. In most patients, the pattern of altered steroids was similar between the primary diagnosis and time of progressive disease. However, we could detect three or more elevated steroids in only 20 of 89 patients with recurrent/progressive disease (22.5%), whereas this was the case in 88% of patients at the primary diagnosis. Of note, tumour burden was quite different (mean tumour size at the time of primary diagnosis was 129 mm vs. 82 mm at the detection of a recurrent or progressive disease; *p* < 0.001). There was no single case in which steroids were elevated that were preoperatively within the normal range.

In 25/89 cases, no steroid hormones were elevated although a recurrence or a progressive disease was confirmed by radiologic imaging, whereas the remaining 44/89 cases either had only 1–2 elevated steroids or the altered hormone pattern was only detected at the time when the progress was also documented by imaging.

In 12/32 (37.5%) patients, steroid hormones, in most of the cases only 11-deoxycortisol, were elevated without any recurrence or progressive disease. However, in only 5/89 patients (5.6%) with recurrent or progressive disease an “endocrine progress” (defined by at least 3 elevated steroids) was falsely diagnosed.

Finally, patients with an early detection of a recurrence or a progression were compared to patients without reliable elevated steroid hormones. We saw again a significant difference in the documented tumour mass in patients with or without an early detection (11.4 cm vs. 7.4 cm; *p* < 0.05; Table 2). Furthermore, mitotane could have an influence on steroid elevation since one third of patients without a clear steroid elevation had a mitotane plasma level above 14 mg/L, whereas this was only the case in 10% with an earlier “endocrine progress” (Table 2).

## 4. Discussion

Despite the fact that several reviews and international guidelines recommend steroid measurements during follow-up of patients with ACC, this is—to our knowledge—the first study investigating the utility of serum LC–MS/MS steroid profiling for the detection of recurrent or progressive disease in patients with ACC. We could demonstrate that in almost a quarter of cases, “endocrine progress” could be detected within a median of 32 days earlier than imaging diagnosed progressive disease. We defined upfront that we counted, as “endocrine progress”, only cases in which at least 3 of 13 adrenal steroids were elevated. With this approach, the number of false-positive samples was as low as 5%. Thus, our study clearly suggests that endocrine follow-up is complementary to imaging. If this result is confirmed by another study, one can even imagine that in a subset of patients (e.g., those with highly elevated hormones prior treatment) regular steroid profiling could be performed to trigger imaging and therefore prolonging the interval between two imaging procedures.

Until now, there are several studies about the use of serum LC–MS/MS measurements as a diagnostic tool for primary diagnosis of ACC [10,12,14,20]. The only study investigating steroid measurements for detection of a recurrent disease is by Chortis et al. [15]. In this study, the use of GC–MS steroid measurements in 32 patients with a recurrence was analysed. Here, the detection of recurrence by steroid profiling preceded detection by imaging by more than two months in 22% to 39% of patients (depending on the individual judgment of three independent investigators). Thus, our results, with an earlier detection in 22.5% of cases, are clearly in line with this study. However, our serum method has at least two main advantages: collecting a single serum sample is much less cumbersome for the patient and the applied LC–MS/MS method is much more widely available in comparison to GC–MS.

One factor influencing the increase of steroid levels could be tumour burden since patients with earlier “endocrine progress” had a significantly higher tumour mass at the time of recurrence or progression in comparison to patients in whom endocrine work-up did not detect progressive disease earlier. Along the same line, at least 3 adrenal steroids were elevated in more than 85% of patients at primary diagnosis—a time when tumour burden was significantly higher than at the time of recurrent or progressive disease. Similar to our findings, tumour mass seemed to influence steroid increase in the study by Chortis et al. since steroid elevation was higher at primary diagnosis in patients with higher tumour mass compared to a lower steroid increase at recurrence in patients with lower tumour volume [15]. Another factor influencing steroid measurement could be the activity of the CYP3A4 enzyme [42]. Although mitotane therapy per se seemed not to decrease the likelihood of detecting an early “endocrine progress”, in patients with a plasma level of more than 14 mg/L the sensitivity of steroid profiling seemed to be reduced.

We certainly have to acknowledge that our study has several limitations. The main weakness is the retrospective study design. Therefore, we cannot exclude that several confounding variables (e.g., tumour burden, different intervals between the blood samplings, and co-treatment with mitotane) account for differences seen in patients with recurrence and altered hormone profiles. A second obvious limitation is the limited number of patients. Since we could include only patients who had their regular follow-up at our centre, we had to exclude many patients, but in the interest of accurate data this approach seemed adequate. Third, our cut-offs were defined upfront based on clinical experience and are not yet validated. In addition, we cannot definitely explain if and to what extent mitotane therapy might interfere with an early detection of progressive disease by steroid profiling. However, one can speculate that the number of patients with elevated steroids at the time of recurrence or disease progression could be even higher if they were not treated with mitotane. Furthermore, our results may be restricted to steroid profiling by LC–MS/MS and should not be simply interpolated to immunoassays. However, the applied LC–MS/MS method is commercially available and used more and more in several countries.

In conclusion, we could demonstrate in 22.5% of our investigated cases with ACC an earlier detection of recurrent or progressive disease by serum LC–MS/MS profiling. Thus, our findings suggest that serum LC–MS/MS is a reliable tool especially in patients with significant tumour volume. Therefore, our study provides new evidence that the proposal of the international guidelines to also measure adrenal hormones during follow-up of patients with ACC is reasonable. Further prospective studies have to define if this method can be used to adapt the interval for imaging in a subset of patients. Such multicentre trials should now be initiated by international networks like ENSAT and A5.

## Figures and Tables

**Table 1:** Patients’ characteristics.

	Cohort A		Cohort B	
	Without Recurrence *n* = 32	Recurrent Disease *n* = 15	Progressive Disease *n* = 52	*p*
Median age—years (IQR)	49.1 (38.3–56.8)	49.1 (34.2–56.1)	45.6 (33.9–58.3)	0.49
Sex—female, *n* (%)	22 (68.8)	6(40)	36 (69.2)	0.095
Preoperative hormone excess *n* (%) Glucocorticoids (GC) Androgens +/− oestrogen Oestrogen Mineralocorticoids (M) GC + androgens +/− oestrogen GC + androgens +/− M Inactive	5 (15.6) 8 (25) 0 0 5 (15.6) 1 (3.1) 3 (9.4)	5 (33.3) 4 (26.7) 1 (6.7) 0 2 (13.3) 0 3 (20)	20 (38.5) 12 (23.1) 0 1 (1.9) 8 (15.4) 4 (7.7) 7 (13.4)	0.012
ENSAT stage at primary diagnosis—*n* (%) 2 3 4	29 (90.6) 3 (9.4) 0	6 (40) 4 (26.7) 5 (33.3)	6 (11.5) 17 (32.7) 28 (53.8)	<0.001
Resection status at primary diagnosis—*n* (%) 0 1 2 X No primary surgery Data not available Surgical approach—*n* (%) Open surgery Minimally invasive surgery No primary surgery Data not available	26 (81.3) 2 (6.2) 0 4 (12.5) 28 (87.5) 4 (12.5)	15 (100) 0 0 0 12 (80) 3 (20)	18 (34.6) 9 (17.3) 0 14 (26.9) 5 (9.6) 6 (11.6) 32 (61.5) 6 (11.5) 5 (9.6) 9 (17.4)	0.017 0.085
Median primary tumour size—mm (IQR)	85 (62–100)	130 (92.5–176.3)	125 (90–155)	0.002
Median Ki67 index of the primary tumour -% (IQR)	15 (5–23)	30 (19–52)	30 (20–50)	0.002
Concomitant mitotane—*n* (%)	25 (78.1)	10 (66.7)	46 (88.5) *	0.13
Concomitant other therapies Platin-based chemotherapy Other chemotherapy Radiation therapy	0 0 0	10 (66.7) 3 (20) 0	61 (82.4) * 6 (8.1) * 3 (5.8) *	0.31
Median time to recurrence/progress defined by imaging– (days) (IQR) Imaging method—*n* (%) Thoracic and abdomen CT Thoracic CT and abdomen MRI FDG-PET/CT	NA 19 (59.4) 5 (15.6) 8 (25)	360 (230–648) 8 (53.3) 3 (20) 4 (26.7)	303 (139–491) * 32 (43.2) * 29 (39.2) * 13 (17.6) *	0.96 0.09
Tumour burden at time of recurrence/progress—*n* Median number of tumoural sites (IQR) Median sum of tumour diameter (mm; IQR) ≤3 cm *n* (%) 3.1—<10 cm *n* (%) ≥10 cm *n* (%)	NA	1 (1–2) 29 (14–65) 8 (53.3) 6 (40) 1 (6.7)	2 (1–3) * 65 (32–125.5) * 16 (21,6) * 33 (44,6) * 20 (27) *	0.12 0.11 0.078
Median follow-up (months) (IQR)	55.5 (28.3–74.9)	30.8 (20.2–65.3)	23.0 (13.7–39.2) *	0.016

Demographics and clinical characteristics of Cohort A and B. Continuous variables were reported as median with lower and upper quartile (Q1–Q3), whereas categorical variables were reported as numbers and percentages. IQR, interquartile range; *n*, number; mm millimetre; cm centimetre; NA, not applicable, CT, computed tomography; MRI, magnetic resonance imaging; FDG-PET/CT 18-fluoro-2-deoxyglucose (F-18-FDG)-positron emission computed tomography. * Values include 74 cases of 52 patients with a progressive disease.

**Table 2:** Possible factors influencing steroid elevation before recurrence or progressive disease in 89 cases with recurrence and progressive disease during follow-up. *n*: number; mm: millimetre; mg/L: milligram per litre.

	Endocrine Progress Prior to Radiological Progress		
	Yes (*n* = 20)	No (*n* = 69)	*p*
Mean sum of tumour diameter at the time of recurrence/progression, mm	113.8	73.7	0.039
Mean number of tumoural sites at the time of recurrence/progression, *n*	2.5	1.9	0.009
Mitotane therapy at the time of recurrence/progress, *n* (%)	19 (95)	57 (82.6)	0.17
Mitotane blood level >14 mg/L at the time of recurrence/progress, *n* (%)	2 (10)	25 (36.2)	0.034

## Data Availability

ACC is a ultrarare disease and patient privacy is important. However, we are committed to sharing data with all qualified external researchers. Requests have to be sent to the corresponding authors. All data provided are anonymised to respect the privacy of the patients.
